# Effects of Acid Modification on Physicochemical Properties of Soybean and Citrus Dietary Fibers and Their Application in Probiotic-Fermented Soy Protein Gels

**DOI:** 10.3390/gels12060548

**Published:** 2026-06-19

**Authors:** Youxin Yan, Meixin Wang, Yuan Zhang, Ke Zhang, Feng Xue

**Affiliations:** 1 School of Pharmacy, Nanjing University of Chinese Medicine, Nanjing 210023, China; yanyouxin_2003@163.com (Y.Y.); 13089978225@163.com (M.W.); 19707812822@163.com (Y.Z.); 16602586868@163.com (K.Z.); 2 Jiangsu Key Laboratory of Medicinal Substance and Utilization of Fresh Chinese Medicine, Nanjing University of Chinese Medicine, Nanjing 210023, China

**Keywords:** dietary fiber, modification, probiotic fermentation, soy protein, gel properties, microstructure

## Abstract

Dietary fibers are valuable food components with documented health benefits, yet their native compact and highly crystalline structures often result in low water hydration, poor adsorption capacity, and limited bioactivity. Chemical modification offers a promising strategy to overcome these functional limitations by disrupting the dense structure and exposing active groups. This study aimed to investigate the effects of acid modification on the physicochemical properties of soybean and citrus dietary fibers and to evaluate the performance of the modified fibers in probiotic-fermented soy protein gels. Compared with native fibers, modified fibers exhibited reduced particle size, rougher and more porous microstructures, and increased exposure of hydroxyl groups. Consequently, they showed significantly (*p* < 0.05) enhanced hydration capacity (increased by 92–541%), antioxidant activity (increased by 15–65%), cholesterol adsorption (increased by 16–75%), and α-amylase inhibition (increased by 26–62%). When incorporated into soy protein-based gels, the modified fibers, particularly those from soybean, lowered gel pH, increased water holding capacity, gel strength, apparent viscosity, and storage modulus, while reducing strain, indicating improved gel network integrity. These findings indicate that acid modification effectively unlocks the functional potential of dietary fibers, positioning the modified fibers, especially from soybean, as promising prebiotic ingredients for plant-based fermented gel products.

## 1. Introduction

Defined as a group of plant-based carbohydrates, dietary fiber escapes digestion by intestinal enzymes and absorption in the digestive tract, but it is subject to fermentation (partial or complete) in the large intestine [1]. This group primarily consists of oligosaccharides, polysaccharides, lignin, and other related plant metabolites [2]. Consuming sufficient amounts of dietary fiber is crucial for improving digestive function, reducing blood lipid levels, regulating blood sugar, and helping with weight management [3]. A lack of dietary fiber in the daily diet has been linked to an increased risk of gastrointestinal disorders, diabetes, and certain cancers. Given the well-documented health benefits, dietary fiber has drawn growing research interest. It is often incorporated into food matrices to produce fiber-enriched products, offering healthier options for consumers. Dietary fiber carries various functional groups, including hydroxyl, aldehyde, and carboxyl groups, which influence the structure of macromolecules and determine their functional behavior [4]. However, when directly added to food products, these functional groups are often not fully exposed, leading to poor functional performance (e.g., poor solubility and bioactivities) and limiting practical applications. Moreover, the inferior functional properties of dietary fiber can negatively affect the color, flavor, and texture of the final functional foods [5]. Therefore, overcoming the inherent shortcomings of dietary fiber through modification, and thereby expanding its use in the food industry, remains a challenge that needs to be addressed.

Physical, enzymatic, and chemical treatments are commonly used to improve the properties of dietary fiber. These methods promote the hydrolysis of polysaccharides and help expose functional groups [6]. Among them, acid treatment stands out as both effective and easy to perform. As a classical chemical modification approach, acidic modification offers considerable economic advantages. It works by breaking down the lignocellulosic chains within dietary fiber, thereby exposing functional groups (hydroxyl and carboxyl groups) and enhancing adsorption capacity as well as hypoglycemic activity [4]. Earlier studies have shown that acid-modified rice bran dietary fiber exhibits higher oil holding capacity, water holding capacity, and glucose absorption [7]. Other reports indicate that acid-treated flaxseed cake dietary fiber develops looser surface structures and better flow characteristics, along with improved hydration properties and greater adsorption capacity for lipids, cholesterol, and nitrite ions [4]. Despite these advances, comparative studies on how acid modification differentially affects fibers from different plant sources (e.g., legume versus fruit) remain limited. To address this gap, we employed acid modification (chosen for its effectiveness, simplicity, and ability to expose functional groups) to improve the functional properties of dietary fibers from soybean and citrus, representing two different sources.

Citrus fruits are widely utilized in various culinary applications, including juices, smoothies, sauces, bakery products, and desserts, generating approximately 50–60% waste in the form of peels, seeds, segment membranes, and albedo [8]. A particularly promising valorization route for this byproduct stream is the recovery of dietary fiber, which has been associated with multiple health benefits, such as enhanced digestive function, reduced serum cholesterol levels, and a lower risk of cardiovascular disease and type 2 diabetes [9,10]. Another notable source of dietary fiber is derived from soybean processing byproducts. Soybean dietary fiber, obtained from abundant and low-cost soybean residue, comprises cellulose, hemicellulose, lignin, and soybean polysaccharides, all of which contribute to cardiovascular health [11,12]. Soybean and citrus were selected as representative sources because they are among the most abundant and commercially relevant byproducts for dietary fiber production within their respective categories (legume processing and fruit processing). From a structural perspective, soybean and citrus dietary fibers differ markedly in their chemical composition and architecture. Soybean fiber is primarily composed of uronic acids, galactose and arabinose [13]. In contrast, citrus fiber contains substantial amounts of arabinose, galacturonic and galactose [14]. These compositional differences lead to distinct functional group exposures, degrees of crystallinity, and surface morphologies, which may influence their susceptibility to acid modification and subsequent functional performance.

Given the potential of both citrus and soybean fibers, the present study selected these two types to investigate the effects of acid modification on their physicochemical properties.

In recent years, the addition of dietary fiber to yogurt has garnered substantial research attention. For instance, apple fiber has been used to modify the texture, structure, and stability of stirred yogurt [15]. Similarly, corn fiber has been incorporated to improve the texture, water-holding capacity, and firmness of low-fat yogurt [16]. Fiber derived from cabbage outer leaves has also been shown to enhance the bioactive properties of yogurt [17]. The inclusion of dietary fiber not only improves the nutritional value but also positively influences the texture and stability of the final product [5]. Nevertheless, most previous studies have concentrated on animal-based yogurt systems [18,19,20], whereas plant-based alternatives have received comparatively little attention.

Recently, the application of probiotics in food products has attracted growing interest, largely owing to their diverse biological activities. These include the alleviation of colitis and constipation, enhancement of immune responses, regulation of gut microbiota, reduction in cancer-related risk, modulation of neurological functions, and lowering of blood glucose and lipid levels [21]. Concurrently, dietary fibers are gaining recognition for their prebiotic properties. They can be selectively fermented by beneficial intestinal bacteria to produce short-chain fatty acids, which confer multiple health benefits [22]. In the context of fermented dairy and plant-based products, the inclusion of dietary fibers has been demonstrated to stimulate the growth and metabolic activity of starter cultures, as well as to enhance the textural and rheological attributes of the resulting gels [15]. Given that probiotic-fermented soy protein gels are emerging as a promising alternative to conventional dairy yogurt, elucidating how acid-modified citrus and soybean fibers affect gel network formation is of considerable scientific and practical relevance.

Therefore, this work aims to apply acid modification technology to dietary fibers from both soybean and citrus sources, exploring the functional and structural changes induced by such treatment. Furthermore, the modified fibers will be incorporated into a plant-based yogurt matrix made from soy protein to evaluate their impact on the gelation properties of soy protein. To the best of our knowledge, no previous study has systematically compared acid-modified soybean and citrus dietary fibers side-by-side, nor has any study evaluated their performance in a probiotic-fermented soy protein gel system. Therefore, the novelty of this work lies in three aspects: direct comparison of acid modification effects on legume-derived (soybean) versus fruit-derived (citrus) dietary fibers; comprehensive evaluation of multiple functional properties (hydration, antioxidant, cholesterol adsorption, α-amylase inhibition) in relation to structural changes; and the application of these modified fibers as prebiotic ingredients in a plant-based fermented gel (soy protein gel) to assess gelation behavior, rheology, and microstructure. This integrated approach not only fills the comparative knowledge gap but also provides practical guidance for selecting fiber sources for plant-based yogurt applications.

## 2. Results and Discussion

### 2.1. Whiteness Index, Repose and Slide Angles of Dietary Fiber

**Figure 1:**
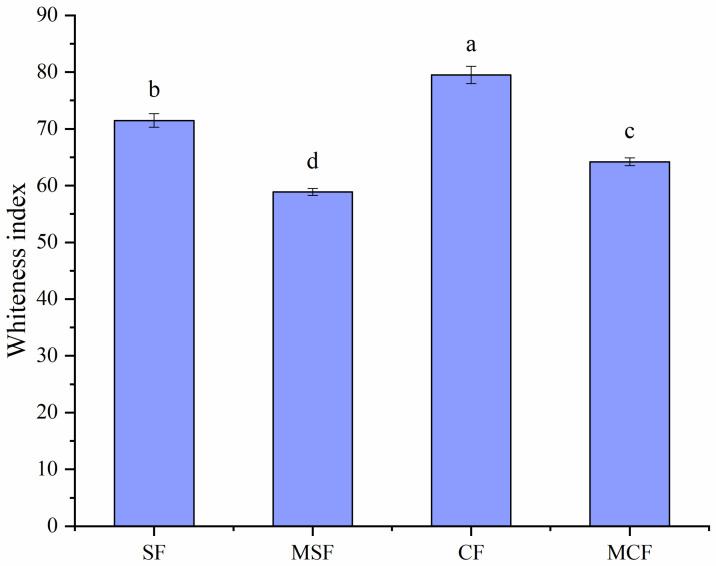
Effects of acid modification on the whiteness index of soybean and citrus dietary fibers. SF, soy dietary fiber; MSF, modified soy dietary fiber; CF, citrus dietary fiber; MCF, modified citrus dietary fiber. Results having different letters within the same pattern are significantly different (*p* < 0.05, *n* = 3).

As illustrated in Figure 1, the whiteness of dietary fibers from different sources varied significantly, with CF exhibiting higher whiteness than that from soybean. Furthermore, the whiteness of the dietary fibers decreased markedly after modification. This reduction may be attributed to the exposure of residual phenolic components originally embedded within the dietary fiber matrix upon modification, which subsequently underwent oxidation, leading to a decline in whiteness [23]. This observation is consistent with previous reports on purple turnip dietary fiber [24] and flaxseed cake dietary fiber [4], where modification resulted in the oxidation of phenolic compounds and a consequent decrease in whiteness. Notably, MSF showed significantly lower whiteness than MCF. This is likely due to the higher initial phenolic content in soybean fiber that is released and oxidized during acid treatment.

**Figure 2:**
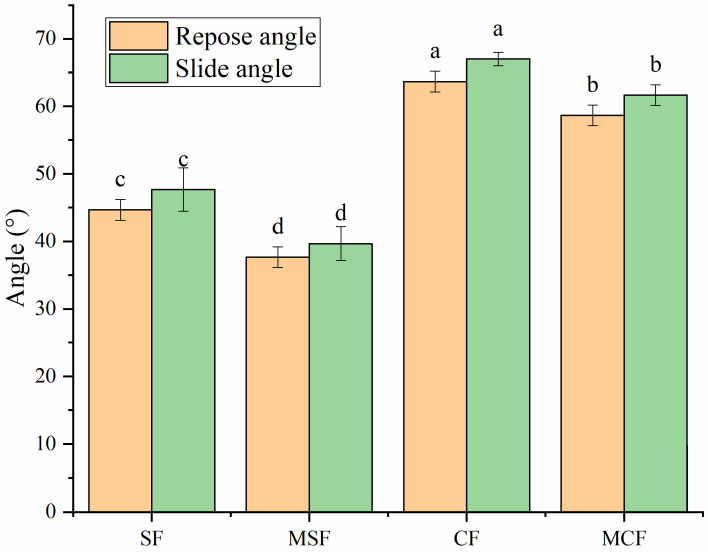
Effects of acid modification on repose and slide angle of soybean and citrus dietary fibers. SF, soy dietary fiber; MSF, modified soy dietary fiber; CF, citrus dietary fiber; MCF, modified citrus dietary fiber. Results having different letters within the same pattern are significantly different (*p* < 0.05, *n* = 3).

As shown in Figure 2, the repose and slide angles of dietary fibers from different sources varied significantly. SF exhibited lower values for both angles compared to CF, suggesting that SF has greater flow characteristics, as a smaller angle of repose and angle of slide indicate lower interparticle friction and cohesion, allowing powder particles to slide or roll past each other more easily [25]. Furthermore, after modification, the angle of repose and slide angle of dietary fibers both decreased notably. This finding aligns with earlier observations on sea buckthorn seed meal dietary fiber [26] and flaxseed cake dietary fiber [4]. In those studies, the authors attributed the reduction to the loose surface structure of the modified dietary fiber, which improves fluidity by diminishing the cohesive capacity of macromolecular substances.

### 2.2. Particle Size of Dietary Fiber

**Figure 3:**
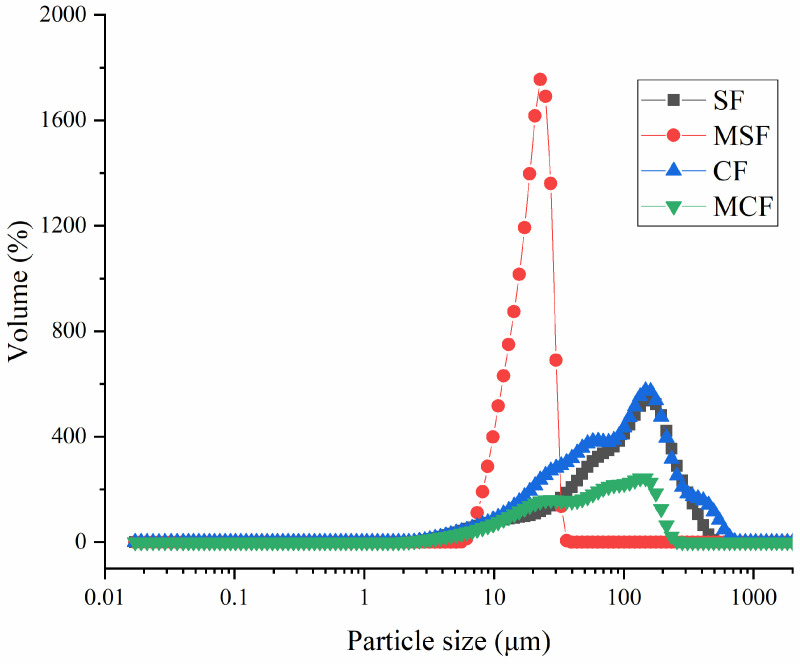
Effects of acid modification on particle size distribution of soybean and citrus dietary fibers. SF, soy dietary fiber; MSF, modified soy dietary fiber; CF, citrus dietary fiber; MCF, modified citrus dietary fiber.

As shown in Figure 3, the particle size distributions of SF and CF exhibited a pattern in which particles larger than 50 μm constituted the predominant fraction. After modification, the size distributions of both fibers shifted toward smaller sizes, indicating that modification reduced the particle size of dietary fibers. Previous studies have reported that modification can disrupt the structure of dietary fibers [27], thereby decreasing their particle size. In addition, it has been shown that modification removes or degrades crosslinking proteins and starch present in the dietary fibers [28], further contributing to the reduction in fiber particle size. Notably, after modification, the particle size distribution of SF became centered at approximately 10 μm, suggesting that the modification had a more pronounced effect on soybean fiber. This difference can be explained by the structural distinctions between the two fibers. Compared to citrus fiber, soybean fiber has a lower initial crystallinity (as evidenced by XRD) and intermolecular interactions (as evidenced by FTIR), which makes it more susceptible to acid-catalyzed hydrolysis. This observation may also imply that the MSF might possess better dispersibility and hydration properties [29].

### 2.3. Fourier-Transformed Infrared Spectroscopy of Dietary Fiber

**Figure 4:**
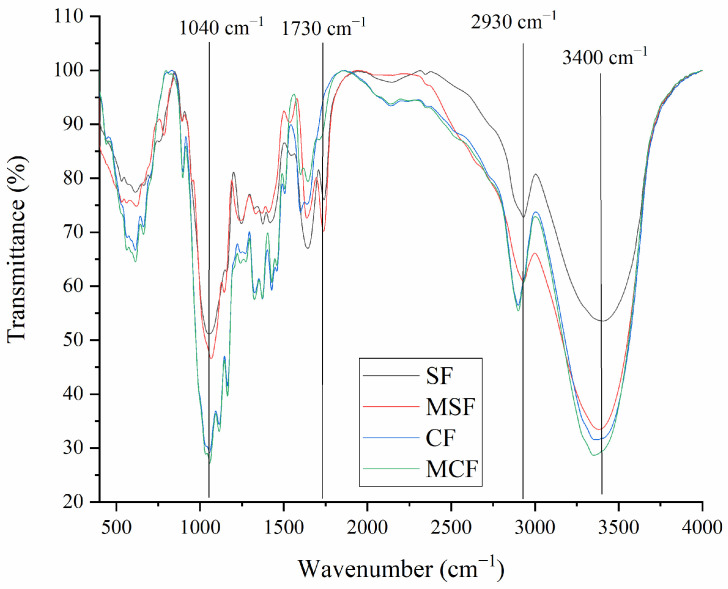
Effects of acid modification on the Fourier-transform infrared (FTIR) spectra of soybean and citrus dietary fibers. SF, soy dietary fiber; MSF, modified soy dietary fiber; CF, citrus dietary fiber; MCF, modified citrus dietary fiber.

Figure 4 shows a broad, intense absorption band centered at approximately 3400 cm^−1^, which mainly originates from the stretching vibrations of –OH groups found in cellulose, hemicellulose, or pectin [30]. After modification, transmittance at this band decreased and a small blue shift was observed, suggesting that hydroxyl groups in the fiber became exposed. This finding aligns with earlier observations on dietary fibers from grapefruit peel [31]. The absorption band observed around 2930 cm^−1^ arises from the stretching vibrations of C–H bonds within the methyl (–CH_3_) and methylene (–CH_2_–) groups of sugar molecules [31]. Notably, this peak appeared at slightly different positions in CF and SF, which could be attributed to their distinct chemical compositions. SF is predominantly composed of uronic acids, galactose and arabinose [13], whereas CF contains a higher proportion of arabinose, galacturonic and galactose [14]. These compositional differences, together with variations in polysaccharide structure and chain conformation, result in distinct chemical environments for the methyl and methylene groups in the two fibers. Consistent with this, the C–H stretching vibration frequencies were observed at slightly different positions in their FTIR spectra. After modification, the band intensity at this position decreased markedly for SF (indicating a substantial reduction in surface hydrophobic groups), whereas only a slight change was observed for CF. A peak appears at 1730 cm^−1^ due to C=O stretching vibrations, most likely attributable to acetyl or esterified pectin groups in hemicellulose [32]. Notably, this peak was clearly present in SF and MSF but was absent in CF and MCF. The origin of this difference remains unclear based on available literature, and further investigation is required. The absorption feature at 1040 cm^−1^ corresponds to both stretching and deformation vibrations of C–O bonds in cellulose, hemicellulose, and lignin. For SF, the transmittance at these peaks decreased markedly after modification, reflecting an increase in hydrophilicity [33]. For CF, only a slight change in transmittance was observed, indicating a less pronounced effect of acid modification on the hydrophilicity of citrus fiber.

### 2.4. X-Ray Diffraction of Dietary Fiber

**Figure 5:**
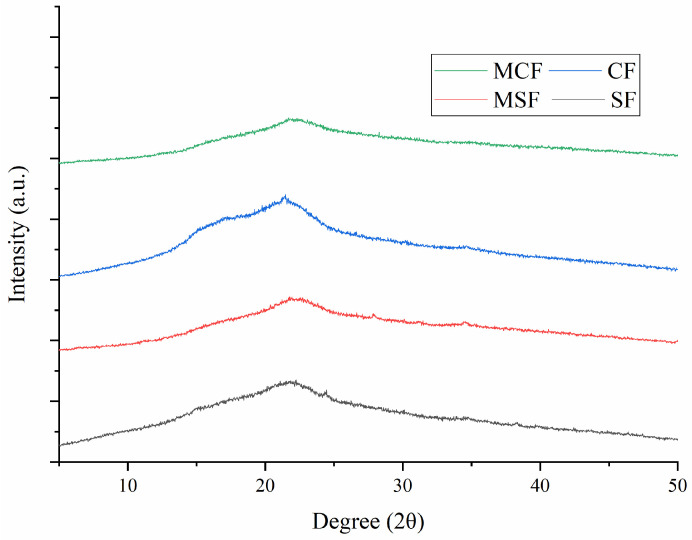
Effects of acid modification on Fourier-transformed infrared spectroscopy of soybean and citrus dietary fibers. SF, soy dietary fiber; MSF, modified soy dietary fiber; CF, citrus dietary fiber; MCF, modified citrus dietary fiber.

As shown in Figure 5, a broad diffraction peak appeared near 2θ = 21° in the native dietary fibers. This peak corresponds to the reflection of cellulose Iβ, which is the dominant crystalline allomorph of cellulose in higher plants [34]. However, in complex dietary fiber samples containing hemicellulose and pectin, the peak may shift to lower angles and broaden due to smaller crystallite sizes and the overlapping contributions from other polysaccharide components [35]. The intensity of this peak decreased markedly after acid modification for both soybean and citrus fibers, accompanied by an increase in the amorphous baseline. A decrease in peak intensity indicates a reduction in crystallinity, as the crystalline fraction of cellulose contributes more strongly to sharp diffraction peaks while the amorphous fraction contributes to a diffuse background [34,35]. These changes can be primarily attributed to the degradation of crystalline regions induced by acid treatment [26], as well as the disruption of intermolecular hydrogen bonding networks within the fiber crystal structure following degradation [36]. Similar observations have been reported in previous studies on flaxseed cake dietary fiber [4] and mulberry-derived dietary fiber [33], where physical or chemical modifications led to an increase in the amorphous region.

### 2.5. Surface Morphology Analysis of Dietary Fiber

**Figure 6:**
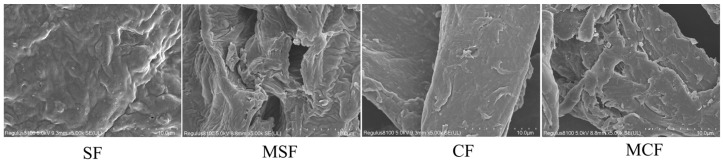
Effects of acid modification on surface morphology of soybean and citrus dietary fibers. SF, soy dietary fiber; MSF, modified soy dietary fiber; CF, citrus dietary fiber; MCF, modified citrus dietary fiber.

As illustrated in Figure 6, SF and CF exhibit relatively compact and smooth surface morphologies. In contrast, the modified fibers display porous, irregular, swollen, and looser structures. Such structural features likely contribute to an increase in specific surface area, thereby enhancing the accessibility of binding sites and functional groups [37]. Previous studies on dietary fibers have similarly reported that modification can disrupt the surface architecture of fibers, leading to exposed hydroxyl groups [38], a looser surface texture [4], and an increased surface area [27]. The structural changes observed in this study (reduced particle size, increased roughness, porous and looser architecture) are in good agreement with previous reports on modified flaxseed cake fiber [4], sea buckthorn fiber [26] and millet bran fiber [39]. These parameters have been consistently correlated with enhanced hydration, adsorption, and gel-reinforcing properties. Importantly, analogous structural features are also exhibited by other gel-forming or texture-modifying agents, such as starch, pectin, gum Arabic, xanthan, carboxymethylcellulose, locust bean gum, and karaya [40]. In each case, small particle size, high surface roughness, and porosity contribute to improved water retention, protein cross-linking, and network stabilization. Thus, the structural parameters identified here are not unique to dietary fibers but represent broadly applicable design features for developing effective gel-enhancing ingredients in plant-based food systems.

### 2.6. Water Holding Capacity (WHC) and Water Swelling Ability (WSA) of Dietary Fiber

**Figure 7:**
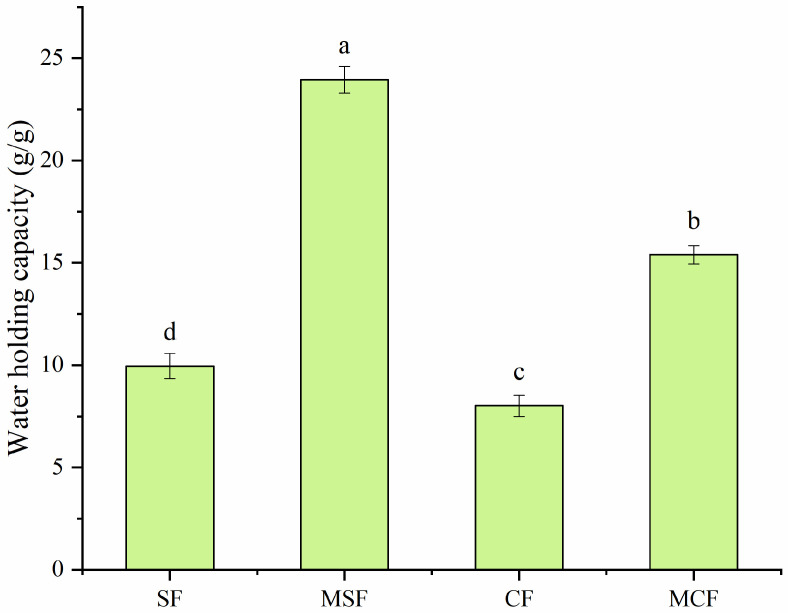
Effects of acid modification on water-holding capacity of soybean and citrus dietary fibers. SF, soy dietary fiber; MSF, modified soy dietary fiber; CF, citrus dietary fiber; MCF, modified citrus dietary fiber. Results having different letters within the same pattern are significantly different (*p* < 0.05, *n* = 3).

**Figure 8:**
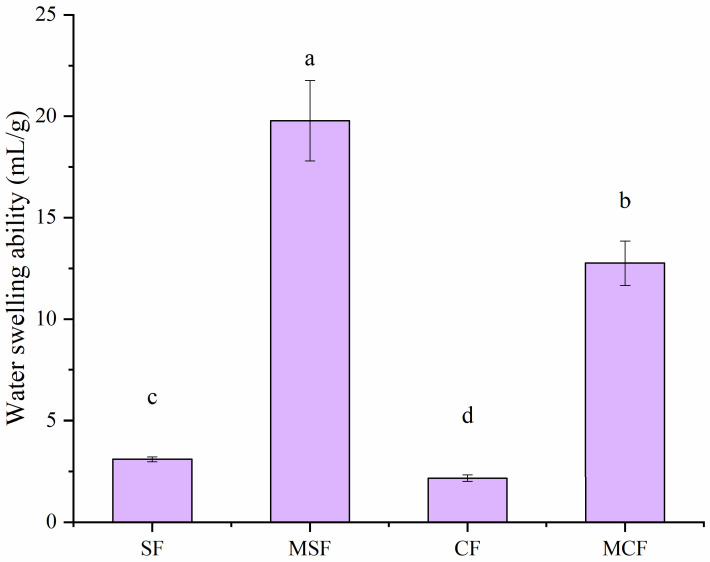
Effects of acid modification on water swelling ability of soybean and citrus dietary fibers. SF, soy dietary fiber; MSF, modified soy dietary fiber; CF, citrus dietary fiber; MCF, modified citrus dietary fiber. Results having different letters within the same pattern are significantly different (*p* < 0.05, *n* = 3).

As shown in Figure 7 and Figure 8, the modified dietary fibers exhibited improved WHC and WSA. This enhancement is mainly attributable to the exposure of hydroxyl groups, which is consistent with the earlier FTIR results. Earlier reports have likewise indicated that acidified dietary fibers possess more hydrophilic side branches, potentially increasing their ability to bind water molecules [41]. In addition, the rougher surface morphology of the modified fibers contributed to better hydration properties, as confirmed by SEM observations. This finding aligns with earlier reports that fibers with coarser surface textures tend to have higher hydration properties [4,7]. Among the samples, MSF displayed the highest WHC and WSA, likely due to its smaller particle size (as shown in Figure 3), which offers a larger specific surface area and further promotes interactions with water molecules.

### 2.7. Antioxidant Activity of Dietary Fiber

**Figure 9:**
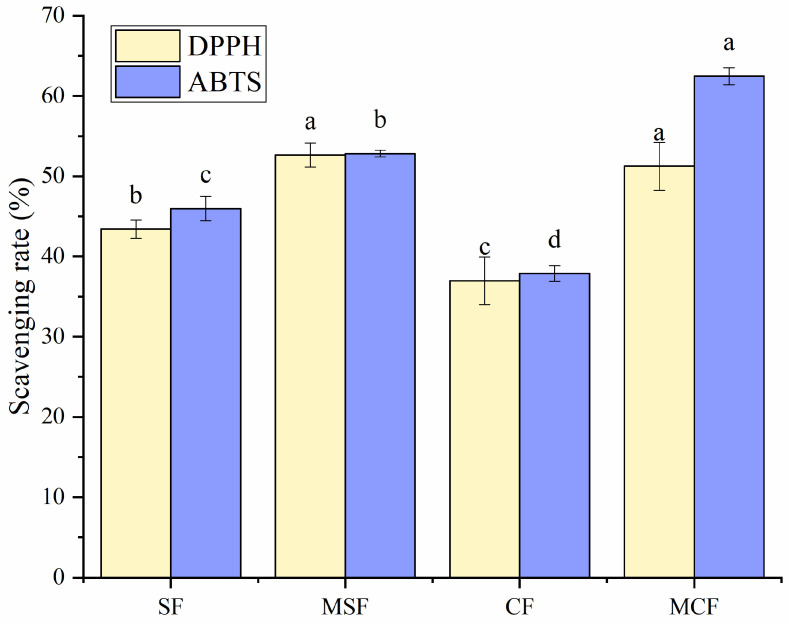
Effects of acid modification on antioxidant activity of soybean and citrus dietary fibers. SF, soy dietary fiber; MSF, modified soy dietary fiber; CF, citrus dietary fiber; MCF, modified citrus dietary fiber. Results having different letters within the same pattern are significantly different (*p* < 0.05, *n* = 3).

Figure 9 shows that all tested dietary fibers possessed free radical scavenging capacity. This can be largely explained by the ability of dietary fibers to donate electrons, which react with free radicals, thereby disrupting the radical chain reaction and producing an antioxidant effect [42]. Moreover, the modified dietary fibers showed a significant increase in their ability to scavenge radicals. This improvement results from the modification-induced structural changes in the fiber, which expose functional groups and thereby enhance scavenging performance [2]. Earlier research has similarly shown that modification promotes the release of numerous hydrogen donors. These donors can provide electrons to neutralize free radicals, thereby efficiently suppressing the radical chain reaction and increasing antioxidant capacity [43]. Additionally, the improved radical scavenging ability resulting from modification may also be ascribed to the porous and rough microstructure of the fibers. Specifically, the roughest and most porous microstructure facilitates the release of polyphenols, thereby further elevating the free radical scavenging capacity of dietary fibers [44]. Research on pea dietary fibers has likewise demonstrated that modified samples possess a porous microstructure, which significantly enhances their antioxidant activity [45].

### 2.8. Cholesterol Adsorption Capacity (CAC) of Dietary Fiber

**Figure 10:**
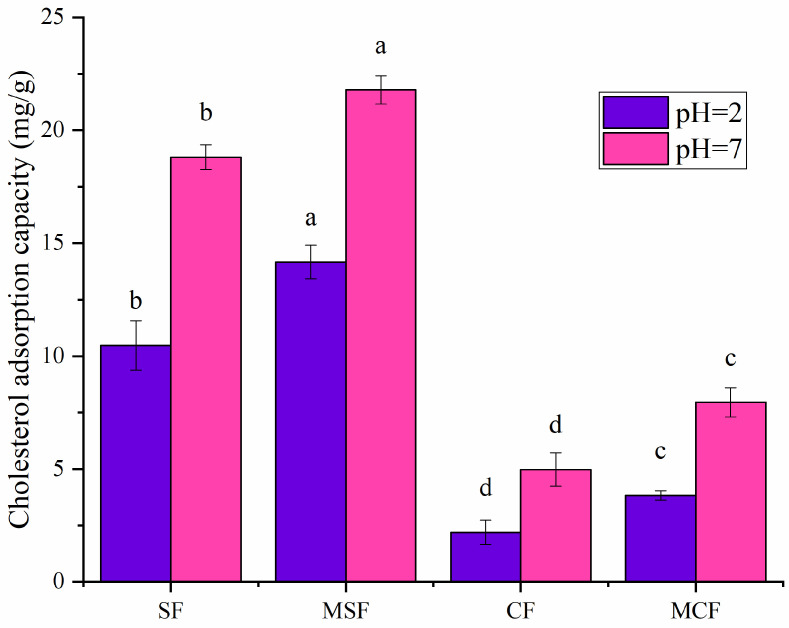
Effects of acid modification on cholesterol adsorption capacity of soybean and citrus dietary fibers. SF, soy dietary fiber; MSF, modified soy dietary fiber; CF, citrus dietary fiber; MCF, modified citrus dietary fiber. Results having different letters within the same pattern are significantly different (*p* < 0.05, *n* = 3).

As shown in Figure 10, under neutral conditions, the CAC of dietary fiber was higher than that under acidic conditions. This observation suggests that dietary fiber exhibits a greater ability to adsorb cholesterol in the intestine than in the stomach, which can be attributed to the dissociation of certain branched-chain groups within the fiber as the pH value increases [39]. Furthermore, the CAC was markedly improved for all modified dietary fibers. This enhancement is likely due to the exposure of functional groups induced by modification, which strengthens the interactions between cholesterol and the fiber chains [46]. Previous research on flaxseed cake dietary fiber has also reported that modification enhances CAC, an effect associated with an increase in ethylene bonds [4]. In addition, the improved CAC may be linked to the loose and porous surface structure of the modified fibers. Earlier studies have demonstrated that such loose and porous microstructures enhance the capillary action on the dietary fiber surface, thereby promoting cholesterol adsorption [47,48]. Notably, under both native and modified conditions, soybean fibers consistently showed greater ability to adsorb cholesterol than their citrus counterparts, suggesting that soybean fiber may contain a greater number of functional groups.

### 2.9. α-Amylase Inhibition Activity (AIA) of Dietary Fiber

**Figure 11:**
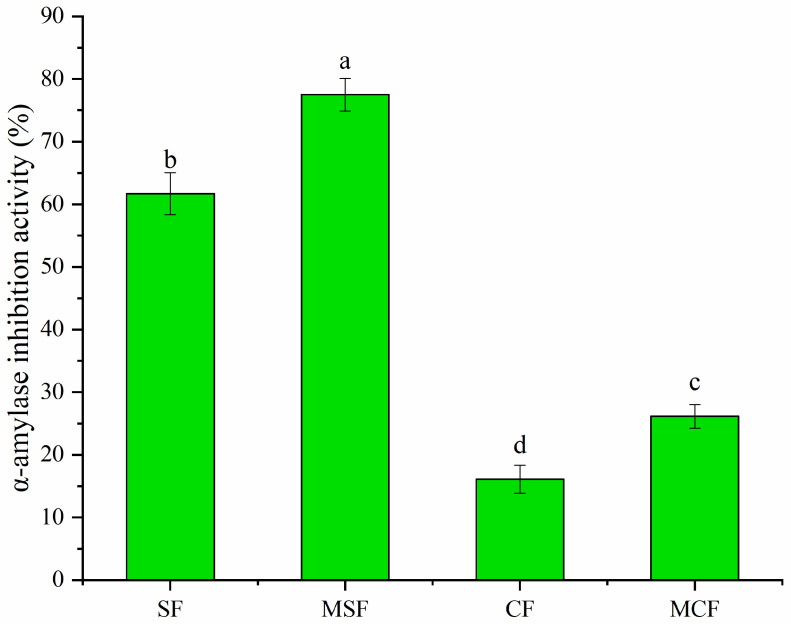
Effects of acid modification on α-amylase inhibition activity of soybean and citrus dietary fibers. SF, soy dietary fiber; MSF, modified soy dietary fiber; CF, citrus dietary fiber; MCF, modified citrus dietary fiber. Results having different letters within the same pattern are significantly different (*p* < 0.05, *n* = 3).

As shown in Figure 11, all dietary fibers exhibited α-amylase inhibitory activity. This can be mainly explained by their ability to reduce enzyme-substrate contact, either by physically entrapping them within the fiber network structure or by increasing the viscosity of food contents in the digestive tract [49]. Moreover, the AIA of the modified dietary fibers was significantly enhanced. This finding is consistent with previous reports on millet bran dietary fiber [27]. This enhancement probably results from the porous surface morphology of the modified fibers, which promotes the attachment of active functional groups to either α-amylase or its substrate, consequently amplifying the inhibition [41]. A more detailed mechanism for the enhanced AIA following acid modification is proposed as follows. First, the reduction in particle size and the development of a rough, porous, and swollen surface microstructure substantially increase the specific surface area of the modified fibers. This provides abundant physical binding sites for both α-amylase and its substrate, facilitating enzyme entrapment and substrate sequestration. Second, FTIR analysis revealed increased exposure of hydroxyl groups and other polar functional groups after modification. These groups can form hydrogen bonds with the active site residues of α-amylase or with the glycosidic bonds of starch, thereby interfering with enzyme-substrate recognition and catalytic efficiency. Third, the modified fibers exhibit significantly higher water holding capacity and water swelling ability, which increases the viscosity of the surrounding medium. Elevated viscosity restricts the diffusion of both enzyme and substrate, thereby slowing down the hydrolysis reaction. Collectively, these synergistic factors account for the significantly improved AIA of acid-modified dietary fibers. Notably, under both native and modified conditions, soybean fibers consistently showed greater ability to inhibit α-amylase activity than their citrus counterparts.

### 2.10. Application in Soy Protein-Based Gel

#### 2.10.1. Physicochemical Properties of Soy Protein-Based Gel

**Figure 12:**
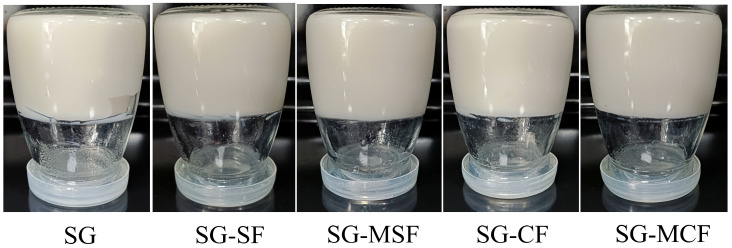
Effect of dietary fiber addition on the appearance of soybean gels. SG-SF: soy protein gel with soy dietary fiber; MSF: soy protein gel with modified soy dietary fiber; CF: soy protein gel with citrus dietary fiber; MCF: soy protein gel with modified citrus dietary fiber.

As shown in Figure 12, soy protein formed a gel after 10 h of fermentation. This is mainly because the organic acids produced during probiotic fermentation lowered the pH to around the isoelectric point of soy protein, thereby inducing protein aggregation and gel formation. This observation aligns with earlier studies documenting the gelation of soy protein induced by probiotic fermentation [50,51]. Notably, in the samples without dietary fiber, clear separation between the gel and the bottle wall was observed, indicating poor gel stability. In contrast, no such separation occurred in the samples containing dietary fiber, suggesting that the addition of dietary fiber enhanced gel stability.

As shown in Table 1, the addition of dietary fiber significantly reduced the whiteness index (WI) of the gels. This reduction may be related to the presence of phenolic compounds in the dietary fiber itself. Previous studies have similarly reported that the incorporation of polysaccharides leads to a decrease in yogurt whiteness, which has been ascribed to the inherent color of these additives [52]. Furthermore, the inclusion of modified fibers resulted in an even greater reduction in gel WI. This phenomenon is likely due to the oxidation of phenolic compounds within the fiber induced by the modification process, a finding that is consistent with the earlier observations on the WI of dietary fibers.

Table 1 indicates that incorporating dietary fiber led to a marked decrease in gel pH. This observation suggests that dietary fiber may function as a prebiotic by promoting probiotic fermentation, thereby lowering the gel pH through the production of short-chain fatty acids [53]. Notably, the incorporation of modified fibers further decreased the gel pH, with the gel containing MSF exhibiting the lowest pH value. This effect may be attributed to the reduction in fiber particle size and the formation of a rougher, looser surface structure after modification, both of which facilitate better utilization of dietary fiber by probiotics. Consistent with prior studies, the inclusion of modified polysaccharides stimulates probiotic fermentation within yogurt [51].

The results in Table 1 indicate that adding dietary fiber greatly improved the water-holding capacity (WHC) of gel. This can be explained by the hydroxyl-rich structure of fibers, which allows them to create stable hydrogen bonds inside the gel matrix at low pH [54]. These interactions contribute to reinforcing the three-dimensional network of the protein gel and effectively prevent whey separation. Comparable findings were documented earlier, in which polysaccharides were likewise observed to significantly improve the WHC of yogurt [55,56]. Furthermore, gels supplemented with modified dietary fiber exhibited higher WHC compared with those containing unmodified fiber, with the MSF-containing gel achieving the highest value among all samples. This outcome is likely because modification exposes functional groups in dietary fiber. On one hand, these groups increase interactions with water molecules; on the other hand, they form a tight network with proteins, thereby enhancing the ability to retain water [51].

According to Table 1, the addition of dietary fiber led to a marked improvement in gel strength. This effect is potentially due to the ability of dietary fiber to facilitate cross-linking between protein molecules, which in turn encourages protein aggregation [57]. In addition, the enhanced gel strength could also result from the metabolism of fiber by probiotics, which leads to increased secretion of exopolysaccharides and further contributes to the improvement of gel strength [58]. In addition, the incorporation of modified dietary fiber led to further increases in gel strength, peaking in the sample with MSF. This is likely because the modified fiber proved more effective at facilitating protein cross-linking and sustaining probiotic metabolic activity [51].

According to Table 1, the addition of dietary fiber led to a significant increase in microbial numbers compared with the control group. This observation suggests that fiber strongly stimulates probiotic proliferation, providing an explanation for the previously noted changes in gel pH. Previous studies have confirmed that probiotics can utilize dietary fiber to support their own growth [59]. In addition, the modified fiber-containing samples showed further increases in microbial counts, among which the gel with MSF reached the highest level. These enhancements stem from the decreased particle size and reduced aggregation of the dietary fiber after modification, together with its increased hydrophilicity and larger surface area. Such alterations likely improve the chances of contact between fiber and probiotics, thus speeding up microbial hydrolysis and resulting in stronger prebiotic effects [60,61]. This increase in viable counts provides direct evidence that both native and modified dietary fibers exert prebiotic activity by supporting the growth of the probiotic starter culture. Notably, the modified fibers, especially MSF, showed a significantly higher prebiotic effect compared to their native counterparts, likely due to their reduced particle size, increased surface area, and enhanced hydrophilicity, which facilitated microbial accessibility and utilization. However, we acknowledge that the present study did not comprehensively characterize the prebiotic properties of the fibers. For instance, the production of individual short-chain fatty acids was not quantified, nor was the selectivity index of the fibers toward specific probiotic strains. Furthermore, the prebiotic effects of modified fibers beyond the fermentation system used here (e.g., in simulated colonic fermentation or in vivo models) remain to be explored. Future studies should therefore include targeted metabolomics of short-chain fatty acids, determination of prebiotic indices, and assessment of structure–prebiotic activity relationships to fully elucidate the prebiotic mechanisms of both native and modified dietary fibers. Comparing the two fiber sources, gels containing modified soybean fiber consistently outperformed those with modified citrus fiber in terms of WHC, gel strength, and microbial count, indicating that MSF is a more effective prebiotic and gel-strengthening ingredient than MCF in this soy protein fermentation system.

#### 2.10.2. Rheological Behavior of Soy Protein-Based Gel

**Figure 13:**
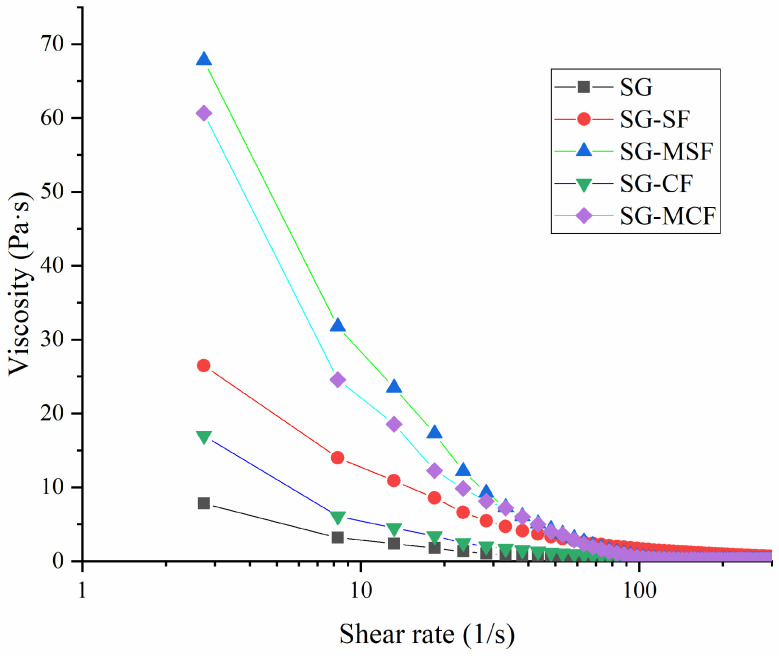
Effect of dietary fiber addition on viscosity of soybean gels. SG-SF: soy protein gel with soy dietary fiber; MSF: soy protein gel with modified soy dietary fiber; CF: soy protein gel with citrus dietary fiber; MCF: soy protein gel with modified citrus dietary fiber.

As demonstrated in Figure 13, the addition of dietary fiber led to a marked increase in gel apparent viscosity compared with the control. This indicates that the fiber served a thickening role. The effect is largely due to the fiber’s ability to facilitate interactions both among protein molecules and between fiber and protein, thus encouraging gel formation and maintaining network stability [52]. Earlier work with polysaccharides has likewise shown that adding them increases yogurt viscosity [62,63]. Moreover, gels containing modified fiber displayed higher apparent viscosity than those prepared with unmodified fiber, with the MSF-supplemented gel showing the highest value. This improvement may be explained by the fact that modification promotes the release of additional functional groups, which are more likely to interact with proteins. For example, the hydroxyl groups liberated during modification could enhance hydrogen bonding between the fiber and proteins, thereby increasing yogurt viscosity [51].

**Figure 14:**
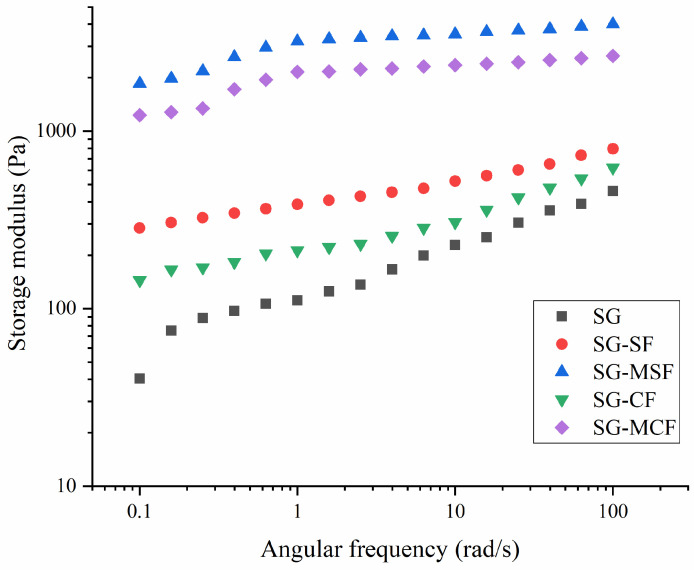
Effect of dietary fiber addition on storage modulus of soybean gels. SG-SF: soy protein gel with soy dietary fiber; MSF: soy protein gel with modified soy dietary fiber; CF: soy protein gel with citrus dietary fiber; MCF: soy protein gel with modified citrus dietary fiber.

As shown in Figure 14, the gels containing dietary fiber displayed an increased storage modulus relative to the control. Moreover, those prepared with modified fiber showed a higher storage modulus than their counterparts made with unmodified fiber, with the MSF-supplemented gel achieving the highest value. This outcome is likely because modification of the fibers helps stabilize the gel matrix and reinforces its overall structural integrity [51].

**Figure 15:**
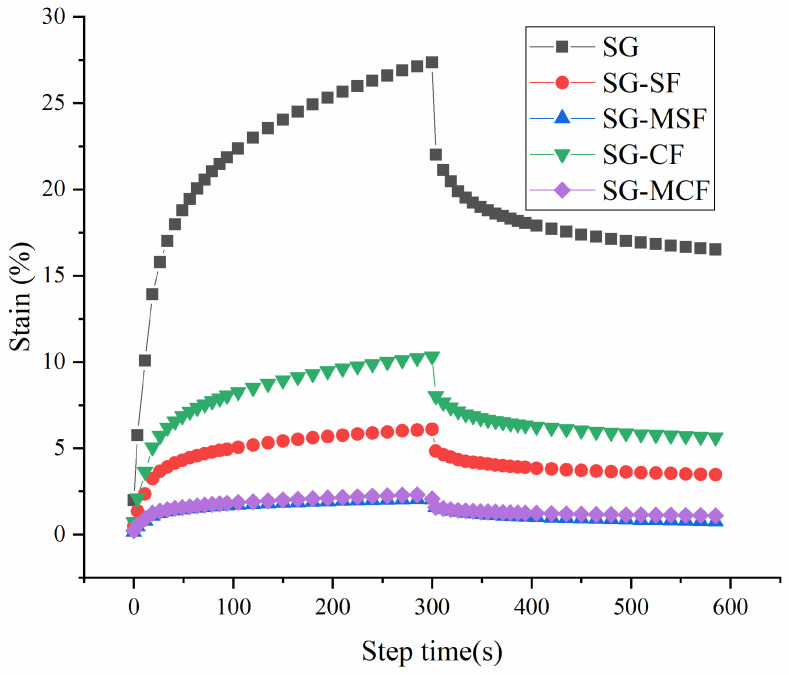
Effect of dietary fiber addition on creep resistance of soybean gels. SG-SF: soy protein gel with soy dietary fiber; MSF: soy protein gel with modified soy dietary fiber; CF: soy protein gel with citrus dietary fiber; MCF: soy protein gel with modified citrus dietary fiber.

As shown in Figure 15, the addition of fiber led to a marked reduction in strain, indicating that fiber-containing gels possessed greater structural integrity. Previous studies have similarly reported that the incorporation of polysaccharides can significantly reduce gel deformation by reinforcing the gel network [51]. Furthermore, lower strain values were recorded for gels prepared with modified fiber than for those containing unmodified fiber. This result further suggests that fiber modification helps strengthen the internal gel network.

#### 2.10.3. Microstructure of Soy Protein-Based Gel

**Figure 16:**
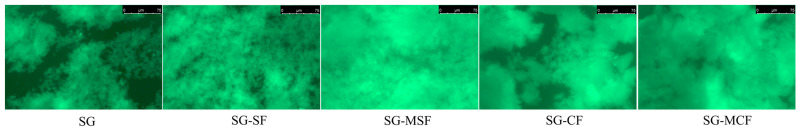
Effect of dietary fiber addition on microstructure of soybean gels. SG-SF: soy protein gel with soy dietary fiber; MSF: soy protein gel with modified soy dietary fiber; CF: soy protein gel with citrus dietary fiber; MCF: soy protein gel with modified citrus dietary fiber. Proteins exhibit green fluorescence upon staining.

As presented in Figure 16, the continuity of the gel structure was markedly enhanced by the addition of fiber. This improvement became even more pronounced when modified fibers were incorporated. Remarkably, in MSF-based gels, most pores disappeared, leading to a tighter and more compressed microstructure. These structural features account for the enhanced water-holding capacity, gel strength, viscosity, storage modulus, and reduced strain observed earlier for MSF-containing gels. Earlier investigations have reported analogous findings, where stronger intermolecular interactions in yogurt fostered a more continuous and compact gel network, ultimately improving the functional properties of the gels [50].

## 3. Conclusions

The present study demonstrated that acid modification effectively altered the structural and functional properties of both soybean and citrus dietary fibers. The modification reduced particle size, disrupted the crystalline region, increased the amorphous content, and converted the smooth surface into a porous, rough, and swollen microstructure, as confirmed by FTIR, XRD, and SEM analyses. Consequently, the modified fibers exhibited superior water holding capacity, water swelling ability, antioxidant activity, cholesterol adsorption capacity, and α-amylase inhibition compared to their native counterparts. The observed improvements in functional properties can be attributed to the increased exposure of active functional groups, the looser and more porous surface architecture resulting from the modification. When applied in probiotic-fermented soy protein gels, the modified fibers, particularly MSF, promoted probiotic proliferation, lowered gel pH, strengthened the protein gel network, and significantly enhanced gel strength, water retention, viscosity, storage modulus, and structural integrity while reducing deformation. Microstructural observations confirmed that the addition of modified fibers eliminated pores and formed a denser, more continuous gel network. This work provides new insights into the valorization of soybean and citrus by-products through a simple chemical modification strategy. The modified fibers, especially from soybean, show great potential as functional prebiotic ingredients for plant-based fermented gel products. From a practical perspective, these findings provide guidance for fiber source selection: acid-modified soybean fiber is particularly suitable for applications requiring high gel strength, water retention, and probiotic proliferation.

Several limitations of the present study should be acknowledged. First, dietary fibers from other important botanical sources, such as cereals and pseudocereals, were not included. Second, the organoleptic properties of the native and modified fibers, including the potential development of bitterness resulting from acid treatment, were not evaluated. Bitterness could arise from the release of limonoid aglycones from citrus fiber or the hydrolysis of residual proteins and isoflavone glycosides in soybean fiber. Third, the antioxidant activity of dietary fibers was assessed only by DPPH and ABTS radical scavenging assays. Other physiologically relevant methods, such as oxygen radical absorbance capacity and ferric reducing antioxidant power, were not employed. Future investigations should therefore broaden the comparative scope to include more fiber-rich species, incorporate sensory evaluation to ensure the practical feasibility of modified fibers in food applications, and adopt a broader panel of antioxidant assays to provide a more comprehensive characterization of the antioxidant properties of both native and modified dietary fibers.

## 4. Materials and Methods

### 4.1. Materials

Soybean dietary fiber (purity >90%) was obtained from Gaotang Lufaxinde Biotechnology Co., Ltd. (Gaotang, China), and citrus dietary fiber (purity > 90%) from Henan Liyang Biotechnology Co., Ltd. (Liyang, China). α-Amylase (2000 U/g) was obtained from Beijing Solarbio Science & Technology Co., Ltd. (Beijing, China). Cholesterol (98% purity) was obtained from Sigma (New York, NY, USA). DPPH, ABTS, fluorescein isothiocyanate (FITC), soy protein (90%) and dinitrosalicylic acid (DNS) reagents were supplied by Shanghai Yuanye Bio-Technology Co., Ltd. (Shanghai, China). The starter culture was obtained from Qingdao Kaimaisen Food Technology Co., Ltd. (Qingdao, China). All other reagents, such as hydrochloric acid and sodium hydroxide, were of analytical grade and purchased from Sinopharm Chemical Reagent Co., Ltd. (Shanghai, China).

### 4.2. Modification of Dietary Fiber

The acid modification of dietary fiber was performed according to a previously described method with minor adjustments [64]. In brief, 10 g of dietary fiber was combined with 2 mol/L NaOH and agitated at 60 °C for 1 h. Following filtration, the remaining solid residue was treated with 1 mol/L HCl at 55 °C for 35 min. The resulting mixture was subsequently centrifuged at 4000× *g* for 20 min, washed twice with distilled water, and freeze-dried to yield the final product. Soy dietary fiber and modified soy dietary fiber were designated as SF and MSF, respectively. Likewise, citrus dietary fiber and modified citrus dietary fiber were designated as CF and MCF, respectively.

### 4.3. Physical Properties of Dietary Fiber

The color of the dietary fiber samples was measured using a color difference meter (HunterLab, Reston, VA, USA). The *L*, a*, and b* values were recorded. Whiteness was calculated following a previously described method [51]. The angle of repose was analyzed according to a published procedure with minor adjustments [65]. To measure the angle of repose, a funnel was mounted above a glass Petri dish. Approximately 4 g of sample powder was poured into the filler until the powder reached the filler outlet. Following a 3 min equilibration, the cone height and radius were measured, and the repose angle was calculated. For the angle of slide, a similar procedure with slight modifications was adopted [26]. In brief, 5.0 g of sample powder were placed on a 120 mm-long glass plate. One end of the plate was gradually lifted until the powder began to slide. The vertical height difference at the lifted end (relative to the horizontal surface) and the plate length were measured, from which the slide angle was derived.

### 4.4. Particle Size of Dietary Fiber

A 0.5% suspension of dietary fiber was prepared by dispersing the sample in deionized water. The particle size distribution was then measured using laser diffraction on an LS13320 particle size analyzer (Beckman Coulter, Indianapolis, IN, USA) [51].

### 4.5. Fourier-Transformed Infrared Spectroscopy (FTIR) of Dietary Fiber

Dietary fiber samples from three independent batches (*n* = 3) were used for analysis. For each batch, 2 mg of dietary fiber was ground with potassium bromide in a 1:100 weight ratio. The resulting mixture was then pressed into translucent pellets using a hydraulic press set to 10 tons. FTIR spectra were acquired on an FTIR-7600 spectrometer (Lambda Scientific, Melbourne, VIC, Australia). Spectra were recorded over the range of 400–4000 cm^−1^ at a resolution of 4 cm^−1^, with 32 scans per measurement. After acquisition, all spectra were baseline-corrected using a rubberband correction to eliminate background drift, followed by normalization to the maximum peak intensity to enable relative comparison of peak positions and transmittance changes between samples. Each sample was measured in triplicate, and the averaged spectrum was used for further analysis.

### 4.6. X-Ray Diffraction (XRD) of Dietary Fiber

X-ray diffraction (XRD) patterns were acquired using a SmartLab diffractometer (Rigaku, Tokyo, Japan) with Cu Kα radiation (λ = 1.540 Å). Each specimen was placed onto a glass slide and lightly pressed to achieve a flat surface. Diffraction intensities were recorded over a 2θ range of 5–50° at a scanning rate of 10°/min, with a slit width of 0.5 mm. The X-ray source was operated at 40 kV and 60 mA throughout the measurement.

### 4.7. Scanning Electron Microscopy (SEM) of Dietary Fiber

For microstructural observation, the dietary fiber sample was coated with a gold layer via sputtering under the following conditions: accelerating voltage 5 kV, sputtering current 15 mA, sputtering time 60 s, working distance 40 mm (target-to-sample distance), sputtering working pressure 5 Pa, and base pressure < 0.1 Pa. Subsequently, the sample was examined at a magnification of 5000× using a SEM (Regulus8100, Hitachi, Tokyo, Japan) with imaging parameters: accelerating voltage 5 kV, chamber pressure < 10^−3^ Pa, emission current 10 μA, working distance 8–10 mm (sample-to-lens distance).

### 4.8. Water Holding Capacity (WHC) of Dietary Fiber

The water holding capacity (WHC) was measured according to an established protocol [66]. In short, 0.5 g of the sample was combined with 20 mL of deionized water and allowed to rest at 25 °C for 30 min. The mixture was subsequently centrifuged at 4000× *g* for 20 min, and the supernatant was removed. The remaining pellet was weighed, and the WHC value was then calculated.

### 4.9. Water Swelling Ability (WSA) of Dietary Fiber

The water swelling ability (WSA) was evaluated by placing 1.0 g of fiber (designated as M) into a graduated tube containing 40 mL of deionized water [4]. The tube was then left at 25 °C for 24 h. The sample’s initial volume prior to swelling was noted as V_1_, and the volume after water uptake was recorded as V_2_. The WSA value was then calculated using the following formula: WSA (mL/g) = (V_2_ − V_1_)/M.

### 4.10. Antioxidant Activity of Dietary Fiber

The antioxidant capacity of the dietary fiber samples was assessed using a slightly modified version of a previously described method [51]. In brief, 1 g of fiber was homogenized in 100 mL of either 50% ethanol or an aqueous solution containing 2.45 mM ammonium persulfate. Subsequently, 0.5 mL of each homogenate was combined with 2 mL of either 0.2 mM DPPH or 7 mM ABTS solution, respectively. The resulting mixtures were incubated in darkness at 37 °C for 30 min, then centrifuged at 5000× *g* for 20 min at 4 °C. The absorbance of the supernatant was read at 517 nm for the DPPH assay and at 734 nm for the ABTS assay using a Spark 10M microplate spectrophotometer (Tecan, Männedorf, Switzerland).

### 4.11. Cholesterol Adsorption Capacity (CAC) of Dietary Fiber

The cholesterol adsorption capacity (CAC) was measured using a previously reported method with minor modifications [67]. Fresh egg yolk (20 mL) was diluted with deionized water (180 mL) and stirred. A 1.0 g aliquot of the sample was added to 25 mL of the egg yolk solution, and the mixture was incubated for 2 h at two different pH levels: pH 2.0 and pH 7.0. After incubation, centrifugation was performed at 5000× *g* for 20 min. The supernatant’s absorbance was then read at 550 nm. The concentrations of cholesterol and cholesterol acyl carrier protein (CAC) were determined via the o-phthalaldehyde method.

### 4.12. α-Amylase Inhibition Activity (AIA) of Dietary Fiber

The α-amylase inhibition activity (AIA) of dietary fiber was assessed using a slightly modified version of a previously reported method [68]. In brief, 50 μL of sample (1% dietary fiber solution) was combined with 100 μL of α-amylase solution (2 U/mL) in phosphate buffer (0.1 M, pH 6.9). The mixture underwent pre-incubation at 37 °C for 30 min. To start the enzymatic reaction, 100 μL of 1% soluble starch solution was added, followed by another 10 min of incubation. Then, 100 μL of dinitrosalicylic acid (DNS) reagent was added to terminate the reaction. Immediately thereafter, the reaction mixture was heated in a boiling water bath for 10 min and subsequently cooled under running tap water. Any loss in volume was made up for by distilled water. Absorbance was read at 540 nm. The percent inhibition was calculated with the following equation: Inhibition (%) = [(A_(blank)_ − A_(sample)_)/A_(blank)_] × 100, where A_(blank)_ is the absorbance of the control (without sample) and A_(sample)_ is the absorbance of the tested sample.

### 4.13. Application in Soy Protein-Based Gel

#### 4.13.1. Preparation of Soy Protein-Based Gel

Soy protein-based gels were produced via gelation induced by probiotic fermentation, using a slightly modified version of our earlier protocol [51]. In brief, soy protein was dissolved in deionized water to prepare a 5% protein solution. Sucrose (5%) and dietary fiber (0.5%) were then added. The mixture was refrigerated at 4 °C overnight to allow full hydration. Subsequently, the solution was homogenized with a high-pressure homogenizer (Ningbo Xinzhi Biotechnology Co., Ltd., Ningbo, China) at 30 MPa for three cycles, followed by sterilization at 95 °C for 15 min. After cooling to 42 °C, 0.3% starter culture was inoculated into the sterilized solution. The inoculated samples were transferred into sterile containers and fermented at 42 °C for 10 h. After fermentation, the gels were placed in a refrigerator and kept at 4 °C for 24 h for post-ripening. The control gel prepared without dietary fiber was designated as SG. Gels containing soy dietary fiber and modified soy dietary fiber were named SG-SF and SG-MSF, respectively, while those containing citrus dietary fiber and modified citrus dietary fiber were named SG-CF and SG-MCF, respectively.

#### 4.13.2. Physicochemical Properties of Soy Protein-Based Gel

The appearance of the gels was recorded using a digital imaging system (Huawei Mate 70, Huawei, Shenzhen, China) for visual documentation. Color parameters, including whiteness, were measured and calculated with the same device following the procedure described earlier. After homogenizing each gel sample, the pH was measured using a calibrated pH meter (Shanghai Yidian Scientific Instrument Co., Ltd., Shanghai, China). The gel samples were then centrifuged at 10,000× *g* for 10 min, and the WHC was computed following a method reported earlier [51]. Gel strength was assessed with a texture analyzer (Stable Micro Systems, TA-XT2i, Godalming, UK). A 100 mL gel sample was transferred into a glass cylinder (50 mm inner diameter) and compressed to half its original height by a cylindrical probe (36 mm diameter) moving at 1 mm/s. The maximum force (in grams) recorded during probe travel was taken as the gel strength. The total viable microbial count in the gels was determined using a previously described protocol [51]. Briefly, each sample was serially diluted with sterile saline, and aliquots of suitable dilutions were plated onto agar media. All plates were incubated for 48 h at 37 °C (BD23 incubator, Binder, Tuttlingen, Germany) before colonies were counted.

#### 4.13.3. Rheological Behavior of Soy Protein-Based Gel

An HR-1 rheometer (TA Instruments, Crawley, UK) fitted with a 60 mm parallel plate geometry was used to measure the rheological properties of the gel samples. Steady shear testing was conducted by increasing the shear rate linearly from 0.1 to 300 s^−1^ at 25 °C, and the corresponding apparent viscosity values were recorded. Dynamic oscillatory measurements were performed over an angular frequency sweep of 0.1–100 rad/s at a constant strain amplitude of 0.1% and a temperature of 25 °C, monitoring the storage modulus (G′). Creep and recovery experiments were also carried out under a constant shear stress of 5 Pa at 25 °C, following a previously described procedure [51]. A steady stress was imposed for 300 s, after which the recovery phase was tracked for an additional 300 s.

#### 4.13.4. Microstructure of Soy Protein-Based Gel

To visualize the protein component in the gel matrix, 1 g of fresh gel was thoroughly combined with 10 μL of FITC solution (0.1 mg/mL in dimethyl sulfoxide). The stained samples were subsequently observed under a fluorescence microscope (DM2500, Leica, Wetzlar, Germany) at 200× magnification.

### 4.14. Statistical Analysis

Data are presented as the mean ± standard deviation (SD) of three independent replicates. Statistical significance between groups (*p* < 0.05) was evaluated using one-way analysis of variance (ANOVA) followed by Tukey’s post hoc test, conducted with SPSS Statistics (Version 25.0, IBM Corp., Armonk, NY, USA).

## Figures and Tables

**Table 1:** Effect of dietary fiber addition on physicochemical properties of soy protein-based gel.

Samples	Whiteness Index	pH Value	WHC (%)	Gel Strength (g)	Microbial Count (10^7^ CFU/mL)
SG	79.88 ± 0.35 ^a^	4.52 ± 0.06 ^a^	62.00 ± 1.83 ^e^	22.25 ± 2.03 ^d^	0.99 ± 0.09 ^d^
SG-SF	76.18 ± 0.30 ^b^	4.34 ± 0.04 ^b^	73.33 ± 1.84 ^c^	30.68 ± 0.14 ^c^	1.68 ± 0.10 ^c^
SG-MSF	74.03 ± 0.38 ^c^	4.12 ± 0.06 ^d^	88.89 ± 4.82 ^a^	38.67 ± 0.65 ^a^	2.88 ± 0.16 ^a^
SG-CF	76.64 ± 0.27 ^b^	4.38 ± 0.03 ^b^	68.89 ± 1.73 ^d^	29.35 ± 1.60 ^c^	1.57 ± 0.09 ^c^
SG-MCF	74.98 ± 0.28 ^c^	4.22 ± 0.02 ^c^	81.94 ± 3.47 ^b^	35.12 ± 2.07 ^b^	2.50 ± 0.11 ^b^

SG-SF: soy protein gel with soy dietary fiber; MSF: soy protein gel with modified soy dietary fiber; CF: soy protein gel with citrus dietary fiber; MCF: soy protein gel with modified citrus dietary fiber. Results having different letters within the same column are significantly different (*p* < 0.05, *n* = 3).

## Data Availability

The raw data supporting the conclusions of this article will be made available by the authors on request.

## Abbreviations

The following abbreviations are used in this manuscript:

FTIR	Fourier-transformed infrared spectroscopy
XRD	X-ray diffraction
SEM	Scanning electron microscopy
WHC	Water holding capacity
WSA	Water swelling ability
CAC	Cholesterol adsorption capacity
AIA	α-amylase inhibition activity
